# Diagnostic approach to interstitial pneumonias in a single centre: report on 88 cases

**DOI:** 10.1186/1746-1596-7-160

**Published:** 2012-11-26

**Authors:** Dirk Theegarten, Heike Maria Müller, Francesco Bonella, Jeremias Wohlschlaeger, Ulrich Costabel

**Affiliations:** 1 Institute of Pathology and Neuropathology, University Hospital Essen, University of Duisburg-Essen, Hufelandstrasse 55, Essen, 45147, Germany; 2 Department of Pneumology/Allergology, Ruhrlandklinik - University Hospital Essen, University of Duisburg-Essen, Tüschener Weg 40, Essen, 45239, Germany

## Abstract

**Background:**

Interstitial pneumonias (IP) cover a broad spectrum of diseases. Open lung biopsies reveal histological patterns and suggest possible diagnoses. Complete clinical records are necessary for final diagnoses. Especially idiopathic interstitial pneumonias (IIP) according to the ATS/ERS classification can only be diagnosed under these predictions. The aim of this study was to compare the results of histological evaluations with the final diagnosis after interdisciplinary case evaluation.

**Patients and methods:**

88 patients with interstitial pneumonia that underwent open lung biopsies were investigated. Histology and clinical records were available for review. Diagnosis was made in three steps: first on the sole basis of histology, second with clinical information given initially and third, on the basis of an interdisciplinary case evaluation.

**Results:**

63 patients (72%) were diagnosed as idiopathic interstitial pneumonias according to ATS/ERS criteria. Further 10 (11%) cases of hypersensitivity pneumonitis, 7 (8%) Langerhans cell histiocytosis and 8 (9%) interstitial pneumonias of other known causes or associations were detected. Histological patterns alone agreed with the final diagnosis in 67%. In 82% histology and clinical information given to the pathologist could provide correct diagnosis. In the rest of cases, especially in non idiopathic interstitial pneumonias, an interdisciplinary case evaluation was needed.

**Conclusions:**

Diagnosis of interstitial pneumonias by open lung biopsies needs sufficient clinical information. Because of the overlap of histological patterns, an interdisciplinary case evaluation that includes at least one clinical expert and one pathologist with excellent expertise and the follow-up of the patients is necessary to find correct diagnosis in all cases.

**Virtual slides:**

The virtual slides for this article can be found here: http://www.diagnosticpathology.diagnomx.eu/vs/5031706258025129

## Background

Diffuse interstitial lung diseases (ILD) are disorders with a large spectrum of possible underlying causes. Most of ILD belong to the group of idiopathic interstitial pneumonias (IIP). But these diagnoses can only be made after exclusion of known etiological factors or associations.

Pulmonary fibrosis was first described by VON BÜHL in 1872 [1]. The first generally accepted classification of idiopathic interstitial pneumonia was introduced by LIEBOW in 1975 [2]. He distinguished usual interstitial pneumonia (UIP), bronchiolitis obliterans with interstitial pneumonia (BIP), desquamative interstitial pneumonia (DIP), lymphocytic interstitial pneumonia (LIP) and interstitial giant cell pneumonia (GIP). In 1990, KITAICHI described a further group designated as “unclassified interstitial pneumonia” [3]. This led to a revision of the Liebow classification by A. KATZENSTEIN [4]. The categories UIP and DIP remained, but LIP and GIP were abandoned, because they were no longer regarded as idiopathic disease. Respiratory bronchiolitis with interstitial lung disease (RBILD), acute interstitial pneumonia (AIP) and the non specific interstitial pneumonia (NSIP) were introduced as new entities.

The ATS/ERS (American Thoracic Society/European Respiratory Society) international multidisciplinary consensus classification of idiopathic interstitial pneumonias was developed in 2002 by a team of clinicians, pathologists and radiologists in order to standardize classification and achieve a broad acceptance among the participating disciplines [5]. By this classification LIP was reintroduced, but non idiopathic cases have to be excluded faithfully [6]. In clinical practise overlap to follicular bronchiolitis has been found [7].

Revised evidence-based guidelines for diagnosis and management of IPF as a collaborative effort between the ATS, ERS, Japanese Respiratory Society (JRS), and Latin American Thoracic Association (ALAT) have been published in 2011 [8]. Non idiopathic origin of IIP has to be evaluated carefully, pulmonary symptoms can even proceed manifestations of connective tissue diseases [9].

Interobserver variability in the diagnosis of ILD is a problem for chest physicians, radiologists and pathologists [10-13]. Discordances also exist between general and pulmonary pathologists in the diagnosis of interstitial lung disease [14]. Therefore, standardization of the diagnostic process and quality assessment are necessary.

The aim of this study was the evaluation of patients with IIP undergoing open lung biopsies in three diagnostic steps to investigate the benefit of clinical information and final interdisciplinary case evaluation.

## Patients and methods

### Selection and categorization of the patients

All cases with histological diagnosis of interstitial pneumonia or pulmonary fibrosis involving patients of the Ruhrlandklinik Essen – West German Lung Center at the University Hospital Essen between 1993 and 2000 were retrospectively selected from the archive of the Department of Pathology at the Ruhr-University Bochum. The indication to perform open lung biopsies had been made by the clinicians on the basis of the available guidelines at that time. All patients have given written consent to surgical procedures und scientific evaluation of data. Data protection was done according to legal foundations. Patients without open lung biopsy (OLB) were excluded. Clinical records were reviewed in each case, follow up was requested by contacting local practitioners. Digital images or photographs of thoracic imaging (CXR, CT and HRCT) were only partially available for diagnostic review and written descriptions of radiological findings were often of poor quality. Therefore results of thoracic imaging were not included in the evaluation process.

### Bronchoalveolar lavage

BAL was performed during local anesthesia using fiberoptic bronchoscopy according to established guidelines [15]. In brief, a flexible bronchoscope was wedged into a segmental bronchus of the middle lobe or the lingula. Sterile isotonic saline was instilled in five to ten 20 ml aliquots up to a total volume of 100–200 ml, with immediate aspiration by gentle suction after each aliquot. The recovered BAL fluid was immediately processed in the laboratory. The fluid was pooled, filtered through two layers of gauze, and centrifuged at 500 g for 10 min at room temperature. The cells were counted in a haemocytometer. Slides were stained with May-Grünwald-Giemsa stain (Merck, Germany) and a total of 600 cells were counted for the cell differentials. A trypan blue exclusion test was performed for evaluating cell viability.

### Histology and immunohistochemistry

Slides were stained with hematoxylin and eosin in all cases, further slides with Elastica van Gieson, Periodic Acid Schiff and Prussian Blue stains were available in parts. In cases of suspected Langerhans cell histiocytosis immunohistochemistry with monoclonal antibodies against S-100 Protein and CD1a was done (ABC method, DAKO Hamburg, Germany). Additionally in one case of LIP staining for lymphocyte subtypes (CD 3 and CD 20, ABC method, DAKO Hamburg, Germany) was performed.

### Diagnostic evaluation process

**Figure 1:**
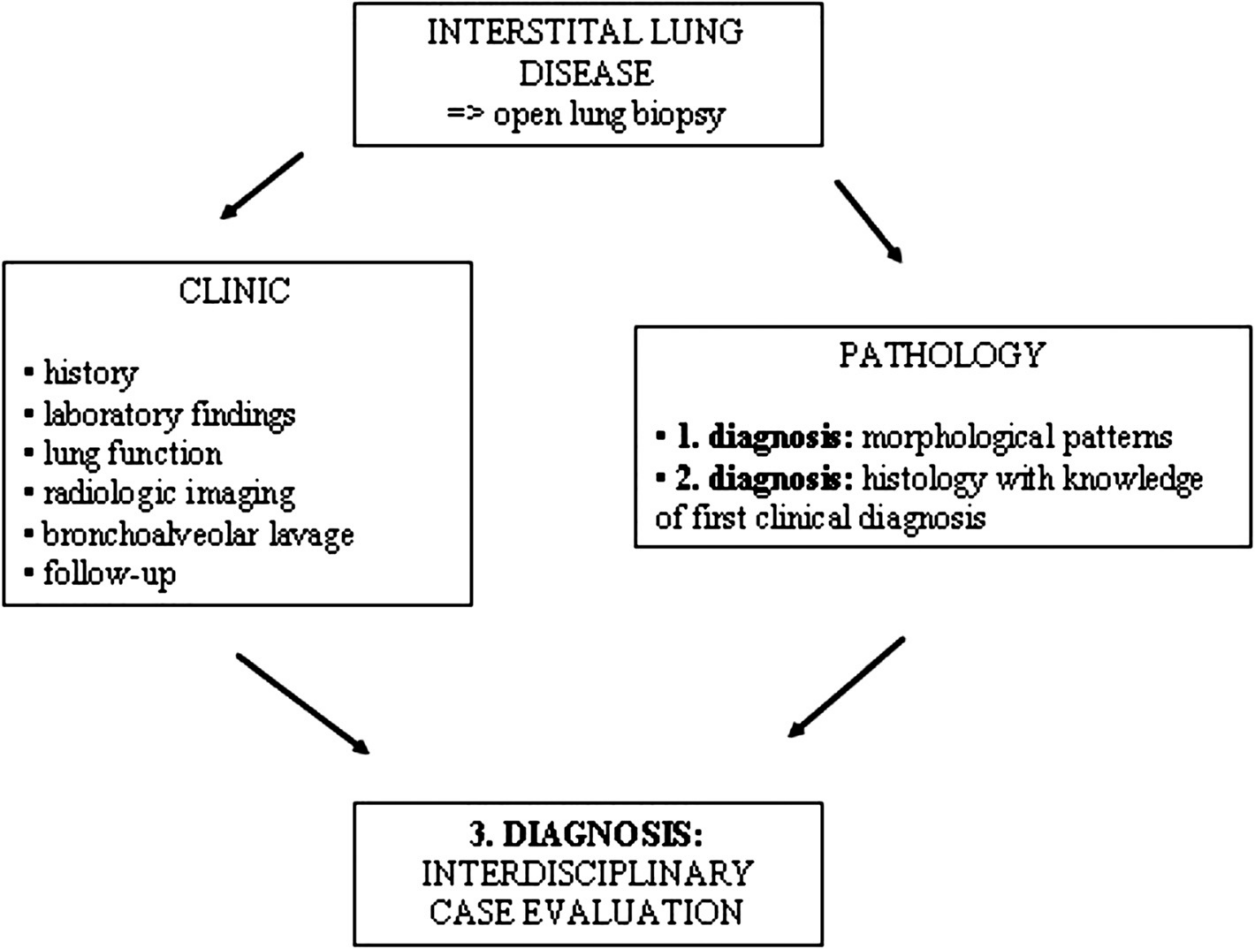
**Diagnostic steps in interstitial lung diseases.** Different steps necessary for sufficient diagnosis.

Diagnostic evaluation was done in three steps. Firstly, diagnosis was made as a slide review on the basis of histological patterns found by the pathologist (DT), blinded to the BAL results and other clinical data. Confidence of histological diagnosis was estimated as certain, probable or possible. If no clear cut diagnosis could be made, the most probable diagnosis was listed. Secondly, histological re-evaluation was done on the basis of clinical diagnoses made by the clinician given on the pathology request form. Thirdly, final diagnosis was made by the pulmonologist (UC) and the pathologist (DT) on the light of all clinical, radiological and histological data as interdisciplinary case evaluation (Figure 1). The results of these three diagnostic steps were compared.

### Statistics

Data are expressed as mean ± standard deviation. We used t-student test and ANOVA, for two or multiple groups respectively, to compare normally distributed variables. Comparison of non-normally distributed variables between two groups was done with the Mann–Whitney *U* test. Comparison of categorical variables between two groups, including the results of the single diagnostic steps vs interdisciplinary case evaluation, was done by the Fisher’s exact probability test. All statistical analyses were done using SPSS version 17.0 for Windows (SPSS Inc., Chicago, IL). Differences were considered statistically significant when the p value was < 0.05.

## Results

A total of 92 cases with interstitial pneumonia underwent diagnostical evaluation. 3 patients with putride pneumonia and one patient with bronchiolitis were excluded from further analysis. Overall confidence of histological evaluation was estimated as certain in 67% of cases (N = 62), probable in 24% of cases (N = 22), and possible in 9% (N = 8). Histological patterns alone agreed with the final diagnosis in 67% of cases and in 82% histology and clinical information provided the diagnosis, compared with interdisciplinary case evaluation (Table 1, p < 0.037 exact Fisher’s test).

**Table 1:** Diagnosis of the different IP subtypes in 3 steps

**Interstitial lung diseases (n = 88)**	**Step 1: histology alone (pattern)**	**Step 2: histology + clinical information**	**Step 3: interdisciplinary case evaluation**
Idiopathic interstitial pneumonia or pattern*	**85**	**77**	**63**
IPF/Usual interstitial pneumonia (UIP)	31	29	27
Non specific interstitial pneumonia (NSIP)	21	20	14
Respiratory bronchiolitis with ILD (RBILD)	14	10	6
Desquamative interstitial pneumonia (DIP)	3	3	3
Cryptogenic organizing pneumonia (COP)	13	12	10
Acute interstitial pneumonia (AIP)	1	1	0
Lymphocytic interstitial pneumonia (LIP)	3	3	3
**Non idiopathic interstitial pneumonia**	**3**	**11**	**25**
Hypersensitivity pneumonitis (HP)	0	3	10
Langerhans cell histiocytosis (LCH)	3	7	7
IP of other known causes or associations	0	1	8
*Correctness of diagnosis (total)*	67%*	87%*	*100%*

*p < 0.05 vs interdisciplinary case evaluation (exact Fisher test).

### Idiopathic interstitial pneumonia (IIP)

63 patients (72%) were diagnosed as idiopathic interstitial pneumonias by interdisciplinary case evaluation according to ATS/ERS criteria (Table 1). There were 27 (43%) patients with idiopathic pulmonary fibrosis (IPF), 14 (22%) with non specific interstitial pneumonia (NSIP), 6 (9.5%) with respiratory bronchiolitis with interstitial lung disease (RBILD), 3 (5%) with desquamative interstitial pneumonia (DIP), 10 (16%) with cryptogenic organizing pneumonia (COP) and 3 (5%) with lymphocytic interstitial pneumonia (LIP).

#### Usual interstitial pneumonia (UIP)/Idiopathic pulmonary fibrosis (IPF)

Male patients were dominant (Table 2). The patients’ mean age was 60 years, the oldest among all ILDs. Clinically, dyspnea (96%) and cough (78%) were present. There were 67% smokers with a mean of 17.2 pack years (Table 2). Bronchoalveolar lavage (BAL) showed a lower percentage of lymphocytes (18%) than NSIP, COP, or HP cases (Table 3).

**Table 2:** Demographics and patients’ characteristics according to diagnosis by interdisciplinary evaluation

	**All**	**IPF/UIP**	**NSIP**	**RBILD**	**COP**	**HP**	**LCH**
**Patients** (N)	74	27	14	6	10	10	7
**Gender**,male/female	43/31	21/6	6/8	5/1*	7/3	2/8**	2/5
**Age**, mean ± SE	53 ± 11	60 ± 11	51 ± 14*	44 ± 6*	58 ± 11	52 ± 10	44 ± 12*
**BMI,** kg/m^2^	26 ± 5	28 ± 5	26 ± 4	26 ± 4	26 ± 4	24 ± 3	24 ± 8**
**Smoking history**							
- current/ex (%)	47 (64)	18 (67)	8 (57)	6 (100)*	7 (70)	1 (10)	7 (100)*
- never smokers	27 (36)	9 (33)	6 (43)	0 (0)	3 (30)	9 (90)	0 (0)
**Pack years**, mean	20.4	17	14.5	27	21	n.a.	22.5
**Symptoms**							
- dyspnoea (%)	65 (88)	26 (96)	13 (93)	4 (67)	7 (70)	10 (100)	5 (71)
- cough (%)	54 (73)	22 (78)	9 (64)	3 (50)	8 (80)	8 (80)	4 (57)
**Duration of symptoms**, years	2.2	1.6	1.7††	1.4	0.3††	4.3**	4.2**
**FVC** % pred, mean ± SE	66 ± 15	61 ± 16	61 ± 9	71 ± 7	72 ± 11	57 ± 13	78 ± 19**
**FEV1**, % pred, mean ± SE	66 ± 14	66 ± 15	65 ± 12	77 ± 12	77 ± 13	63 ± 14	54 ± 21†
**TLC**, % pred, mean ± SE	78 ± 20	67 ± 14	77 ± 15	91 ± 15*	85 ± 19*	68 ± 10	98 ± 17*
**PaO2**, mmHg	74 ± 10	73 ± 8	73 ± 8	80 ± 8	70 ± 14	78 ± 10	72 ± 13
**PaCO2**, mmHg	38 ± 4	39 ± 4	36 ± 3	39 ± 3	40 ± 5	39 ± 3	37 ± 4
**AaDO2**, mmHg	28 ± 16	33 ± 19	30 ± 11	13 ± 6**	25 ± 12	22 ± 10	16 ± 7

*n.a*. = not available.

* p <0.005 vs IPF/UIP.

** p < 0.05 vs IPF/UIP.

† p < 0.05 vs RBILD and COP.

†† p < 0.05 vs HP and LCH.

**Table 3:** BAL findings according to final interdisciplinary diagnosis

**BAL counts (%)**	**Normal range**	**UIP/IPF**	**NSIP**	**RBILD**	**COP**	**HP**	**LCH**
macrophages	>84	64 ± 20	45.3 ± 31	77 ± 21*	46 ± 33	33 ± 18**	86 ± 17
lymphocytes	<13	18 ± 13	36 ± 27	14 ± 9	40 ± 33†	60 ± 18††	4.5 ± 3
neutrophiles	<3	10 ± 6	13.3 ± 11	6.8 ± 6.2	8 ±11	5.5 ± 5	7.5 ± 10
eosinophiles	<0.5	7.5 ± 4.7	5 ± 6.5	2 ± 2	5.7 ± 4	1.3 ± 0,8	1.5 ± 2
mast cells	<0.5	0.5 ± 0.4	0. ± 0.3	0.2 ± 0.2	0.3 ± 0.3	0.2 ± 0.3	0.5 ± 0.4
CD4/CD8	1.1-3.5	1.6 ± 1.5	2.1 ± 1.7	0.6 ± 0.1	2 ±0.9	3.7 ± 2.7	n.a.

*n.a*. = not available.

* p < 0.05 vs NSIP, COP and HP.

** p < 0.05 vs UIP/IPF.

† p < 0.05 vs UIP/IPF, HP und RBILD.

†† p < 0.05 vs UIP/IPF, NSIP, COP, RBILD, und LCH.

**Figure 2:**
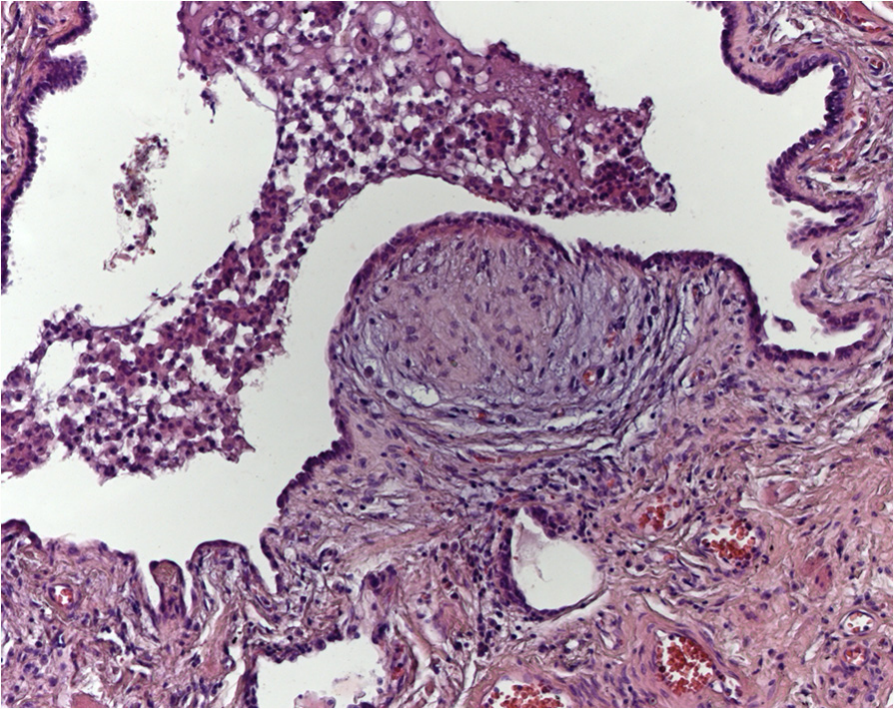
**Usual interstitial pneumonia.** The UIP pattern shows honey combing and fibroblastic foci (haematoxylin eosin stain (HE), original magnification 100x).

Microscopically a UIP pattern with heterogene changes, honeycombing, a variable fibrosis, mucus plugging, lymphoplasmacytic infiltrates of variable densities and fibroblastic foci was seen (Figure 2).

#### Non specific interstitial pneumonia (NSIP)

In this group, most patients were female (Table 2). The mean age was 49.2 years and dyspnea (93%) and cough (64%) were found present since 1.7 years (Table 2). Moreover, 50% of the patients were suffering from clear expectorations and chronic fatigue (data not shown). There were 57% smokers with a mean of 14.5 pack years. Bronchoalveolar lavage showed a mild lymphocytosis (36%) (Table 3).

**Figure 3:**
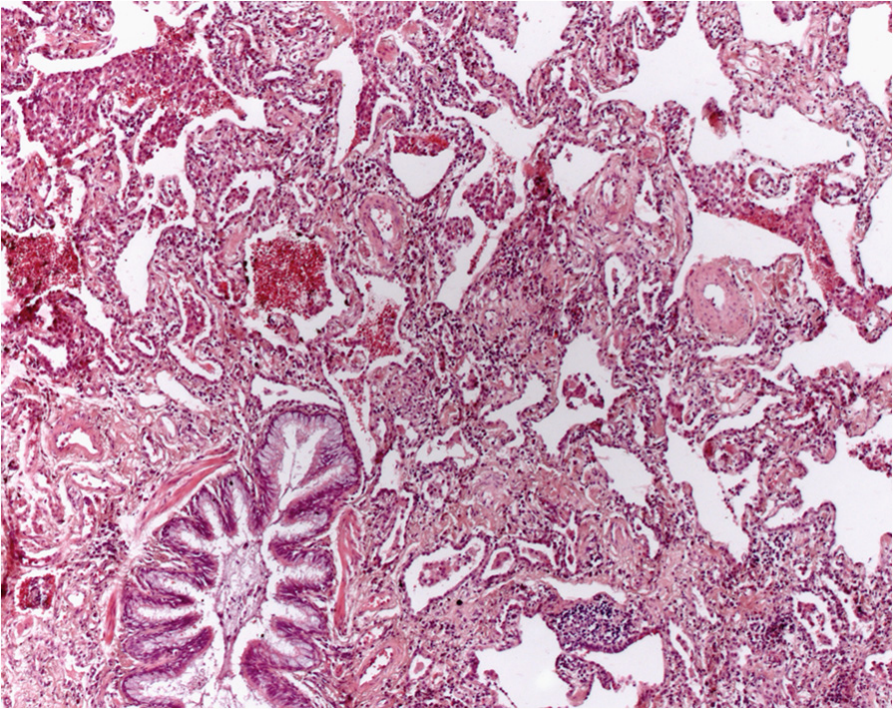
**Non specific interstitial pneumonia.** A mesh-like fibrosis of the alveolar septa is seen. Fibroblastic foci are not found. Aggregates of lymphocytes are sometimes found (HE, original magnification 40x).

Histologically, interstitial pneumonia without any diagnostic criteria of other pulmonary diseases was found. The alterations consisted primarily of a mild to moderate interstitial chronic inflammation, usually with lymphocytes and a few plasma cells. A mesh-like or bronchiolocentric fibrosis sometimes also with honeycombing, but without fibroblastic foci was demonstrated (Figure 3). All cases showed fibrotic changes, however, a prominent inflammation (cellular pattern) could not be noted. NSIP patterns were also found in cases of EAA and interstitial pneumonias of other known causes and associations (Table 1).

#### Respiratory bronchiolitis with interstitial lung disease (RBILD)

Male gender was dominant, in this group (Table 2). Beside dyspnea and cough, expectoration and fatigue were also present (both in 67% of patients). All patients were smokers with a mean of 27 pack years (Table 2). Bronchoalveolar lavage showed a prevalence of macrophages (77%) (Table 3).

**Figure 4:**
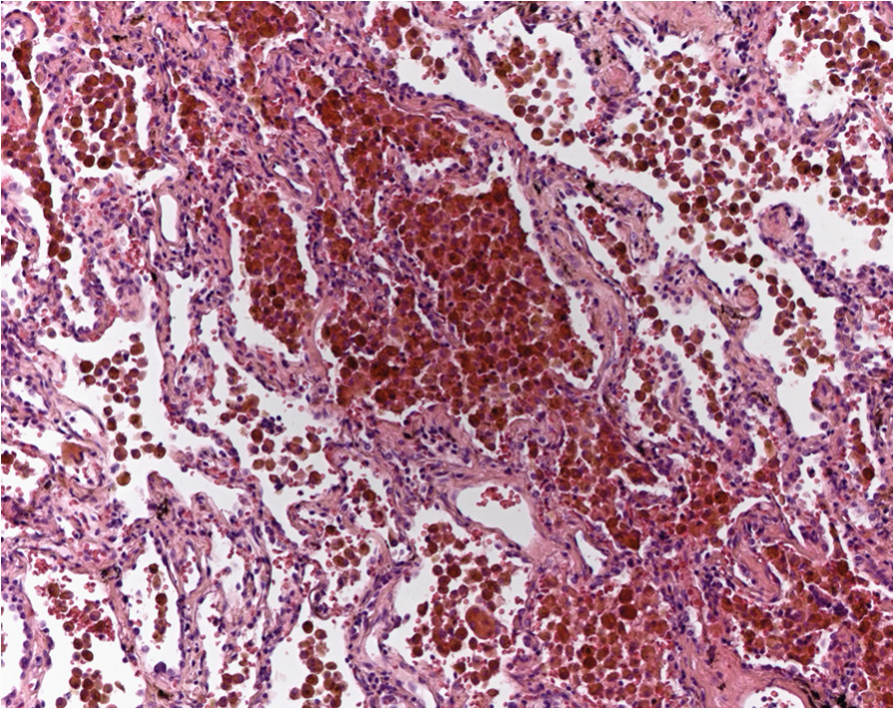
**Respiratory bronchiolitis with interstiatial lung disease.** In RBILD bronchiolocentric aggregates of macrophages and an associated interstitial fibrosis are seen (HE, original magnification 40x).

Morphologically a bronchiolocentric aggregation of macrophages in combination with slight fibrosis and some interstitial infiltrates were seen (Figure 4). Respiratory bronchiolitis was also found in EAA and LH, which could be clarified after immunohistochemistry or case evaluation (Table 1).

#### Desquamative interstitial pneumonia (DIP)

Patients were mostly male (5/6), the mean age was 50.5 years (Table 2). Two patients were active smokers with a mean consumption of 8 pack years. Bronchoalveolar lavage showed an increase of neutrophils (34%) and eosinophils (4%).

**Figure 5:**
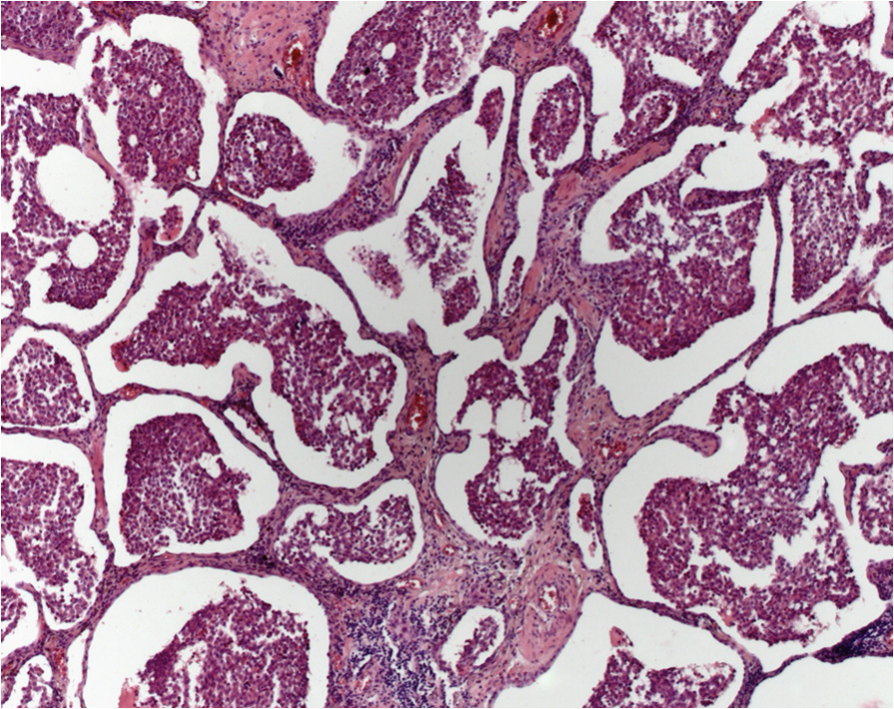
**Desquamative interstitial pneumonia.** A pronounced diffuse intraalveolar aggregation of macrophages and an interstitial fibrosis can be found in DIP (HE. original magnification 40x).

Biopsies revealed a massive intraalveolar aggregation of macrophages. Besides a low grade interstitial fibrosis without honeycombing, some lymphocytes were seen (Figure 5). All cases were diagnosed already without clinical data (Table 1).

#### Cryptogenic organizing pneumonia (COP)

Patients were mainly male and smokers, with a mean of 21.4 pack years (Table 2). Half of the patients had a history of pneumonia (data not shown). In the BAL fluid, lymphocytosis was seen (40%) (Table 3).

**Figure 6:**
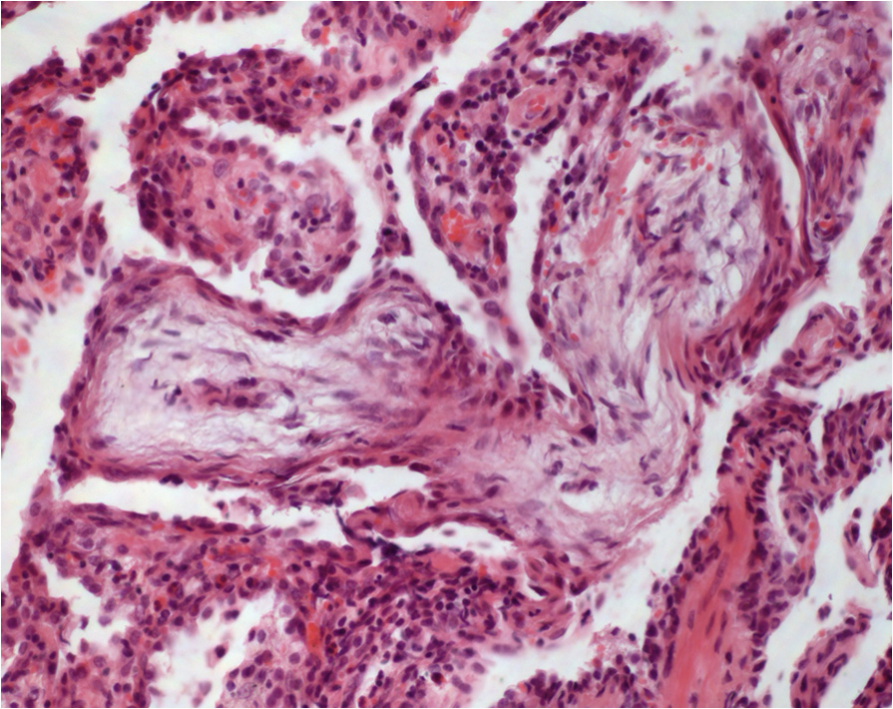
**Cryptogenic organizing pneumonia.** In alveolar ducts and bronchioles buds of granulation tissue are detected (HE, original magnification 100x).

Morphologically granulation tissue was found within the bronchioles, alveolar ducts and the alveolar spaces, which was accompanied by an interstitial inflammatory reaction (Figure 6). Organizing pneumonia (OP) was also seen in cases of NSIP and interstitial pneumonias of other known causes and associations, which caused initial misdiagnoses before case evaluation (Table 1).

#### Lymphocytic interstitial pneumonia (LIP)

All patients (n = 3) were male and in the mean 55.9 years old. There were no smokers among the patients (Table 2). In BAL, lymphocytosis was seen (45.5%).

**Figure 7:**
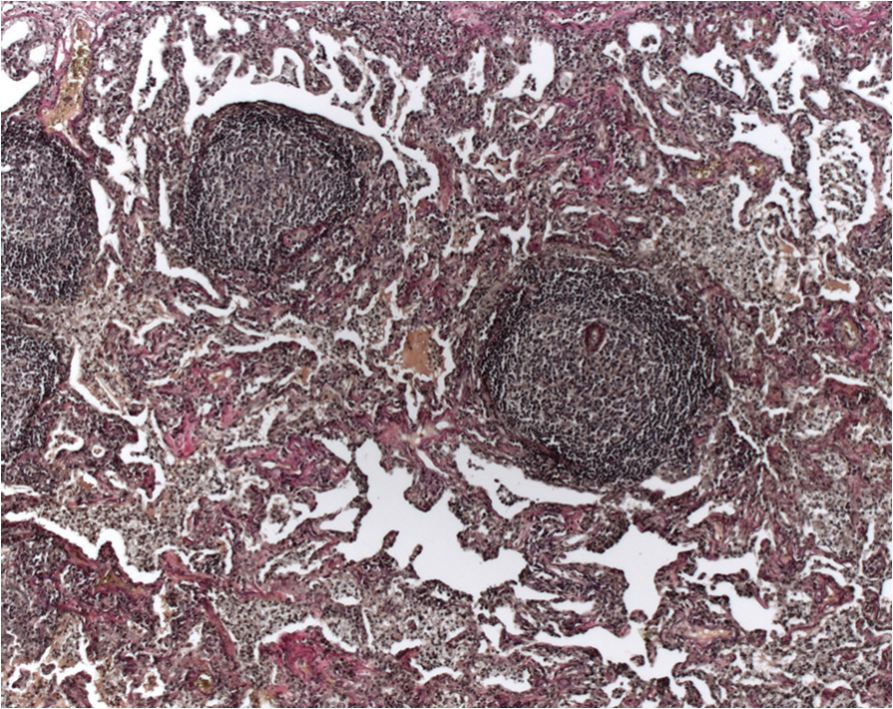
**Lymphocytic interstitial pneumonia.** Prominent infiltrates of lymphocytes and plasmacells and some germinal centers are seen (HE, original magnification 40x).

Microscopically, a dense lympho-plasmacellular infiltration with germinal centres but without blast proliferations were seen (Figure 7). There were no findings suggesting mucosa associated lymphoma. No evidence of clinical or subclinical connective tissue diseases or immunodeficiency was given in these patients. All cases could already be diagnosed without any clinical information (Table 1). Because of the small number of patients enrolled, no statistical calculation could be performed.

### Non idiopathic interstitial pneumonia

25 patients (28%) had interstitial pneumonia of non idiopathic origin. Among these were 10 cases of hypersensitivity pneumonitis (11%), 7 of Langerhans cell histiocytosis (8%), and 8 of interstitial pneumonias of other known causes and associations (9%). Non idiopathic interstitial pneumonias could not be diagnosed without interdisciplinary case evaluation (first step: 12% vs. second step: 44% correctness).

#### Hypersensitivity pneumonitis (HP)

There was a prevalence of females in this group (Table 2). Dyspnea was seen in all patients; cough (80%) and expectorations (70%) were also reported (Table 2). All patients were positive for precipitins to several different avian serum antigens (data not shown). In the BAL 60% lymphocytes, 33% macrophages, 5.5% neutrophils, 1.3% eosinophils and 0.2% mast cells were seen (Table 3).

**Figure 8:**
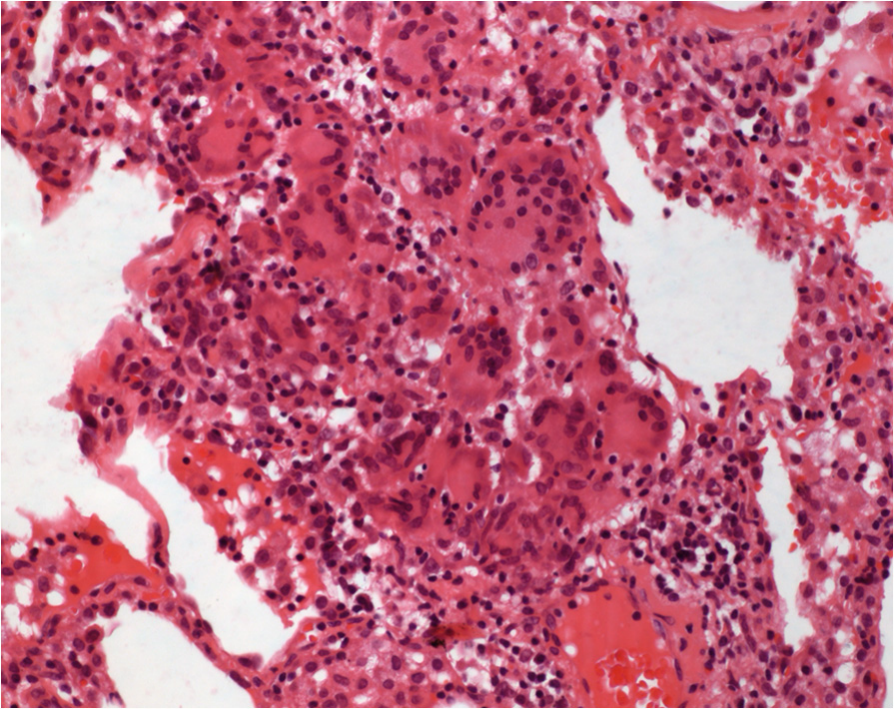
**Hypersensitivity pneumonitis.** Interstitial infiltrates of lymphocytes and plasmacells and some aggregates of histiocytic cells are found (HE, original magnification 100x).

Cases were classified according to dominating pattern as UIP (n = 2), RB (n = 2), NSIP (n = 5) and OP (n = 1). In three cases histiocytic aggregates and granulomas could be found in the second look, which allowed histopathological diagnosis of HP (Figure 8, Table 1). Only case specific evaluation allowed diagnosis in all patients.

#### Langerhans cell histiocytosis (LCH)

These patients were younger than those with IPF, NSIP, and the other ILD (p < 0.05) and were 100% smokers, with a mean consumption of 22.5 pack years. In the BAL 4.5% lymphocytes, 86% macrophages, 7.5% neutrophils, 1.5% eosinophils and 0.5% mast cells were seen (Table 3).

**Figure 9:**
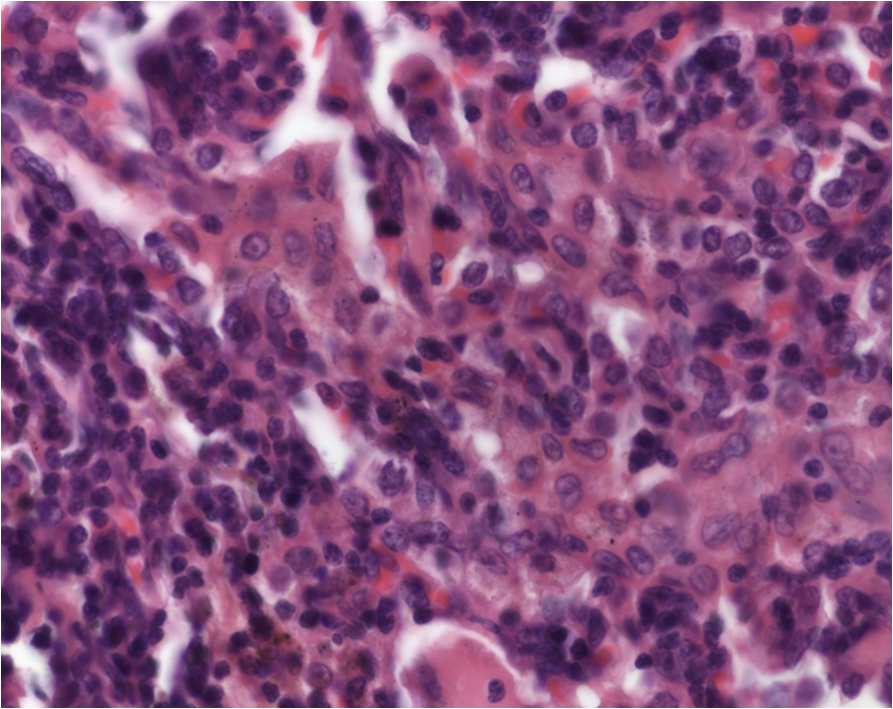
**Langerhans cell histiocytosis.** Patchy infiltrates of histiocytes, lymphocytes and plasmacells are seen (HE, original magnification 100x).

**Figure 10:**
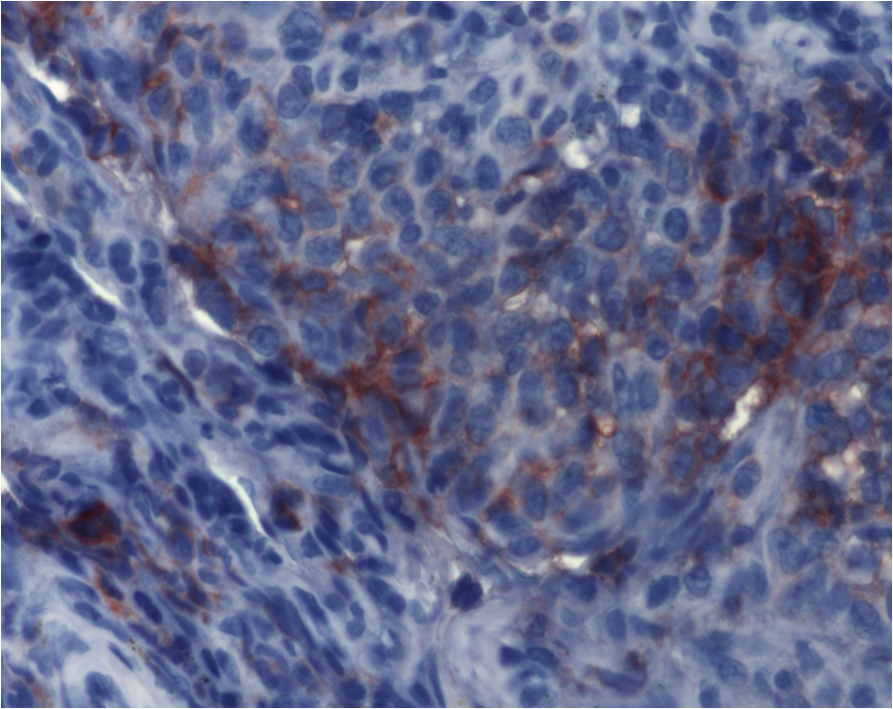
**Immunohistochemistry of Langerhans cell histiocytosis.** Aggregates of Langerhans cells are detectable by monoclonal antibodies against CD1a (ABC-method, original magnification 200x).

Initially without clinical information four cases were diagnosed as RBILD (Table 1). Morphologically definitive diagnosis could be made in all cases by immunohistochemical demonstration of Langerhans cells (positive reaction for CD1a and S100 protein, Figures 9, 10).

#### Interstitial pneumonias of other known causes and associations

There were 8 patients in this group, mostly male (Table 2), their mean age was 55.4 years. Dyspnea was present in all patients, and cough in 62.5% and fatigue in 62.5%. Most patients were smokers (62.5%, mean 13.1 pack years).

Morphologically OP (n = 3) and NSIP-like (n = 2), UIP-like (n = 2) or DAD-like (n = 1) patterns were seen. OP was found in IP with concomitant giant bullous emphysema, colitis ulcerosa and alcoholic cirrhosis. NSIP-like histology was seen in asbestosis and bone marrow transplantation, UIP-like changes in Jo-1 syndrome and after ACE inhibitor therapy. A DAD-like pattern was found in a patient with rheumatoid arthritis (Table 4).

**Table 4:** Patterns in interstitial pneumonias of other known causes and associations (n = 8)

**Histological pattern**	**Total**	**Etiology**
organizing pneumonia	3	Giant bullous emphysema* (1), Colitis ulcerosa (1), Liver cirrhosis (1)
UIP-like	2	Jo-1 syndrome (1), ACE-Inhibitors (1)
NSIP-like	2	Asbestosis (1), bone marrow transplantation (1)
DAD-like	1	Rheumatoid arthritis (1)

*in dominant ILD.

Asbestosis was diagnosed after detection of asbestos body fragments in the tissue and of asbestos bodies in BAL. But all other cases were only detected after case specific evaluation (Table 1).

## Discussion

In general, the diagnostic criteria defined by the ATS/ERS classification of IP were applicable in all cases. Distributions of subgroups of IIP in this study were in accordance with the existing literature (Table 5). Some small differences might be explained by selection of patients for open lung biopsies. The number of LIP cases seems to be higher than in other studies, where it is described as rare disease with a frequency below 2% [16,17].

**Table 5:** Distribution of the 4 main subgroups of IIP in published cohorts

**Author, Country**	**n**	**IPF/UIP**	**NSIP**	**DIP/RBILD**	**COP**
Our study, Germany	60	45%	23%	15%	17%
Bjoraker 1998, USA [18]	91	69.2%	15.4%	11%	4.4%
Nagai 1998, Japan [19]	111	58%	28%	n.a.	14%
Travis 2000, USA [20]	101	55%	29%	16%	n.a.
Nicholson 2000, UK [21]	78	47%	36%	17%	n.a.

n.a. = not available.

With regards to demographics and patients’ characteristics, male predominance in IPF was also found in other studies [22-25], and also in RBILD and DIP [26-28] as well as in COP [29-32]. Concerning NSIP, some studies found a female predominance [33,34], while others noted a male predominance [18,25,35]. In comparison to other studies the clinical history, main clinical symptoms and the rate of smokers in the different subgroups of IP showed only small deviations (Table 6).

**Table 6:** Demographics and clinical symptoms in different subgroups of IIP compared with other studies

**1: Demographics and clinical symptoms in IPF**							
**Author**	**n**	**m : f**	**age**	**dyspnoe**	**cough**	**symptoms (years)**	**smoker**
Our study	27	4.4:1	59.4	96.3%	77.7%	1.7	66.7%
Carrington 1978, USA [23]	53	1.7:1	51	n.d.	n.d.	2.5	71%
Matuso 1996, Japan [24]	30	2.5:1	60	88.6%	100%	1.1	60%
Bjoraker 1998, USA [18]	64	1:1	65	89%	71%	n.d.	54%
Daniil 1999, UK [25]	15	4:1	56	100%	60%	1.5	80%
Nicholson 2000, UK [21]	37	8.3:1	57.2	n.d.	n.d.	1.3	78%
2: Demographics and clinical symptoms in NSIP							
**Author**	**n**	**m : f**	**age**	**dyspnoe**	**cough**	**symptoms (years)**	**smoker**
Our study	14	3:4	49.2	92.9%	64.3%	1.7	57%
Bjoraker 1998, USA [18]	15	1.3:1	57	100%	85%	n.d.	57%
Katzenstein 1994, USA [33]	64	1:1.5	46	n.d.	n.d.	0.7	58%
Park 1996, Korea [34]	7	1:6	56	n.d.	n.d.	0.33	14.3%
Daniil 1999, UK [25]	15	1:1.1	56	100%	60%	1.5	60%
Nicholson 2000, UK [21]	28	2.5:1	53.5	n.d.	n.d.	0.9	64.3%
Cottin 1998, France [35]	12	1:1	52.5	100%	67%	n.d.	50%
3: Demographics and clinical symptoms in RBILD							
**Author**	**n**	**m : f**	**age**	**dyspnoe**	**cough**	**smoker**	**pack years**
Our study	6	5:1	44.6	66.7%	50%	100%	26.6
Myers 1987, USA [26]	6	5:1	36	83.3%	83.3%	100%	39
Yousem 1989, USA [27]	18	1.25:1	36	67%	50%	100%	32
Myers 1992, USA [36]	?	1.7:1	36.1	70.8%	58.3%	100%	33.4
Moon 1999, UK [37]	10	1:1	47.1	70%	30%	90%	39.4
4: Demographics and clinical symptoms in COP							
**Author**	**n**	**m : f**	**age**	**dyspnoe**	**cough**	**smokers**	
Our study	10	7:3	54.8	70%	80%	70%	
Nagai 1998, Japan [19]	16	1:2	56.9	n.d.	n.d.	31%	
Guerry-Force 1987, Canada [29]	15	3:1	56	78.5%	86%	54.5%	
Costabel 1992, Germany [38]	10	7:3	55	90%	90%	n.d.	
Müller 1987, Canada [31]	15	11:4	56.6	78.6%	86.7%	54.5%	
King 1992, USA [32]	112	1.2:1	58	49%	72%	57%	
Izumi 1992, Japan [39]	34	1:1	57	47%	76%	44%	

### Differences in the three different diagnostic steps

Histological patterns alone agreed with the final diagnosis in 67% of the cases. In 82% histology and clinical information could provide correct clinical diagnosis compared with final case evaluation. This provides a significant improvement of correct diagnosis, but only interdisciplinary case evaluation clarified all cases adequately. Strong distinctions are found in the different diseases.

Diagnosis of IPF/UIP based on the histological pattern alone was withdrawn in 2 cases after receiving clinical information and in further 2 cases after interdisciplinary case evaluation. UIP pattern is compatible with the clinical diagnosis of IPF if other etiological factors can be excluded. A questionnaire of 11 centres for pulmonary fibrosis published in Germany 2003, involving 62 patients revealed that IPF had been diagnosed usually 21 months after the beginning of symptoms [40]. Open lung biopsies were taken in 34%, high resolution CT was done in 71% and precipitins were analysed only in 33%. These data point to a relatively poor standard in the diagnosis of IPF and excluding interstitial pneumonias of known origin and association with UIP pattern before the publication of the ATS/ERS statement. Limitations of the clinical criteria for diagnosis of IPF have been demonstrated in other studies [41]. In HRCT honeycombing was seen in 44.4% of our cases with IPF. According to the actual guideline for IPF OLB is no longer necessary in cases with proven IPF in the appropriate clinical and radiological setting (HRCT with UIP pattern). Therefore OLB will now only be done in cases with possible IPF by history and HRCT [8].

RBILD was overdiagnosed by histology plus clinical information compared with final evaluation (10 vs. 6 cases). A variable amount of RB can be found in smokers, which might explain overdiagnosis in those cases. Also in smokers with Langerhans cell histiocytosis RB is usually seen.

Idiopathic NSIP was overdiagnosed by histology plus clinical information compared with final evaluation (20 vs. 16 cases). Diagnosis of non-specific changes is difficult, therefore further evaluation is particularly crucial.

COP was nearly correctly diagnosed by histology plus clinical information compared with final evaluation (12 vs. 13 cases). In one case diagnosis was withdrawn in step 2 because changes were no longer evaluated as adequately expressed. The diagnosis of OP can be difficult if changes are only seen in small parts of the tissue. In this entity, correlation with radiomorphology (patchy consolidation) is essential for the correct diagnosis.

Microscopical diagnosis of DIP and LIP was done without problems and remained the final diagnosis in all steps. The DAD pattern could be diagnosed histologically as well, but proved to be associated with rheumatoid arthritis after case evaluation.

Diagnosis of non IIP without clinical information is difficult. Only 3 of 7 cases with LCH were diagnosed by histology alone. In clinically suspected LCH immunohistochemistry has to be done, which achieved correct diagnosis in all cases. In our cases HP was not suspected by histology alone. With clinical information, histological features of HP were recognized in 3 of 10 patients. In chronic HP a variety of changes ranging from UIP or NSIP patterns to OP is known [42]. In the smoking related diseases RBILD and LCH an overlap of patterns is seen regularly, which is reported in the literature as well [43].

Interstitial pneumonias of other known causes and associations are characterized by morphological patterns also seen in IIP, the only difference is the detection of a known etiological factor [44]. Without carefully evaluated clinical history and laboratory examinations these diseases cannot be diagnosed.

With regards to the BAL findings, we found that differential cytology is of additional value in the diagnostic process. This was also shown in other studies (Table 7). Highest degree of lymphocytosis in IIP is seen in COP. A percentage of over 60% is suggestive for HP. Entities like bronchiolocentric interstitial pneumonia [45], idiopathic bronchiolitis [46-48] or inhaled drug induced ILD [49], have to be excluded histologically and clinically. Further aspects in diagnosis, prognosis and therapy of IP will be offered by systemic biology [50], surfactant expression and immunohistochemistry [51,52], and genetics [53].

**Table 7:** BAL cell counts in different subgroups of IIP in other studies*

**Subgroup**	**Author**	**n**	**lympho.**	**macroph.**	**neutroph.**	**eosinoph.**
**UIP/IPF**	Our study	22**	18.0%	64%	10%	7.5%
	Haslam 1980 [54]	18	3.8%	62.7%	9.8%	4.9%
	Costabel 1992 [30]	22	15%	61%	19%	5%
	Matuso 1996 [24]	30	19.4%	73.1%	4.7%	2.8%
	Shindoh 1986 [55]	20	22.5%	67.9%	7%	2.1%
	Daniil 1999 [25]	8	8.4%	76.8%	9.6%	5.8%
	Nagai 1998 [19]	64	7.2%	83%	5.9%	3.3%
**RBILD**	Our study	5**	14%	77%	6.8%	2%
	Myers 1987 [26]	3	2.3%	95.7%	2%	n.d.
**NSIP**	Our study	14**	36%	45.3%	13.3%	5%
	Katzenstein 1994 [33]	n.d.	37.3%	47.4%	8%	5.5%
	Park 1996 [34]	7	36.5%	34.4%	23.6%	4.8%
	Daniil 1999 [25]	8	9.3%	79.3%	7.8%	3.2%
**COP**	Our study	9**	40%	46%	8%	5.7%
	Nagai 1996 [56]	16	44.4%	45.5%	6.4%	2.2%
	Costabel 1992 [30]	10	44%	39%	10%	6%
	Epler 1994 [48]	12	41%	51%	4%	3%

*mast cells are not mentioned in all studies, therefore total numbers (percentage) do not reach 100% in each study. **results were not available in all cases.

Open lung biopsies are only undertaken in otherwise unclear cases. Histology may show only non characteristic features. Case specific evaluation of clinical and pathological data is therefore a necessity for the exact diagnosis of IP, which was demonstrated by other studies [10,13] and recommended by other experts [17]. Major limitations of our study were the incompleteness of reviewable HRCT data for all patients and the lack of a second pathologist to calculate the level of agreement.

## Conclusions

Diagnosis of interstitial pneumonias, a group of quite rare diseases, requires sufficient clinical information and the knowledge of possible clinical, radiological and histological patterns.

An interdisciplinary case evaluation that includes at least one clinical expert and one pathologist with excellent expertise and the follow-up data of the patients is necessary to find correct diagnoses.

## Abbreviations

ATS: American thoracic society; COP: Cryptogenic organizing pneumonia; CT: Computer Tomogram; CXR: Chest X rays; DAD: Diffuse alveolar damage; DIP: Diffuse interstitial pneumonia; ERS: European respiratory society; FEV1: Forced expiratory volume in 1 second; FVC: Forced vital capacity; HP: Hypersensitivity pneumonia; HRCT: High resolution computer tomography; IP: Interstitial pneumonia; IIP: Idiopathic interstitial pneumonia; ILD: Interstitial lung disease; IPF: Idiopathic pulmonary fibrosis; LCH: Langerhans cell histiocytosis; LIP: Lymphocytic interstitial pneumonia; NSIP: Nonspecific interstitial pneumonia; OLB: Open lung biopsy; OP: Organizing pneumonia; UIP: Usual interstitial pneumonia; TLC: Total lung capacity.

## Competing interests

The authors declare that they have no competing interests.

## Authors’ contributions

DT designed the study, did all histological investigations (first step and second step) and wrote the manuscript. HM collected data from all patients and did statistical analysis. FB revised statistical analysis and the manuscript. JW reviewed the manuscript from the view of pathology. UC did review of clinical and radiological data and suggested final diagnosis. Interdisciplinary case evaluation was done by DT and UC. All authors revised the final manuscript and gave their consent for publication.
